# Evaluating the tumor biology of lung adenocarcinoma: A multimodal analysis

**DOI:** 10.1097/MD.0000000000016313

**Published:** 2019-07-19

**Authors:** Ki Hwan Kim, Seong-Yoon Ryu, Ho Yun Lee, Joon Young Choi, O. Jung Kwon, Hong Kwan Kim, Young Mog Shim

**Affiliations:** a Department of Radiology and Center for Imaging Science, Samsung Medical Center, Sungkyunkwan University School of Medicine, Seoul; b Department of Radiology, Myongji Hospital, Goyang; c Department of Nuclear Medicine; d Division of Pulmonary and Critical Care Medicine, Department of Medicine; e Department of Thoracic and Cardiovascular Surgery, Samsung Medical Center, Sungkyunkwan University School of Medicine, Seoul, Republic of Korea.

**Keywords:** diffusion magnetic resonance imaging, intratumor heterogeneity, lung adenocarcinoma, multimodal analysis, positron emission tomography

## Abstract

We evaluated the relationships among functional imaging modality such as PET-CT and DW-MRI and lung adenocarcinoma pathologic heterogeneity, extent of invasion depth, and tumor cellularity as a marker of tumor microenvironment.

In total, 74 lung adenocarcinomas were prospectively included. All patients underwent 18F-fluorodeoxyglucose (FDG) PET-CT and MRI before curative surgery. Pathology revealed 68 stage I tumors, 3 stage II tumors, and 3 stage IIIA tumors. Comprehensive histologic subtyping was performed for all surgically resected tumors. Maximum standardized uptake value (SUVmax) and ADC values were correlated with pathologic grade, extent of invasion, solid tumor size, and tumor cellularity.

Mean solid tumor size (low: 1.7 ± 3.0 mm, indeterminate: 13.9 ± 14.2 mm, and high grade: 30.3 ± 13.5 mm) and SUVmax (low: 1.5 ± 0.2, indeterminate: 3.5 ± 2.5, and high grade: 15.3 ± 0) had a significant relationship with pathologic grade based on 95% confidence intervals (*P* = .01 and *P* < .01, respectively). SUVmax showed a strong correlation with tumor cellularity (R = 0.713, *P* < .001), but was not correlated with extent of invasion (R = 0.387, *P* = .148). A significant and strong positive correlation was observed among SUVmax values and higher cellularity and pathologic grade. ADC did not exhibit a significant relationship with tumor cellularity.

Intratumor heterogeneity quantification using a multimodal-multiparametric approach might be effective when tumor volume consists of a real tumor component as well as a non-tumorous stromal component.

## 1 Introduction

Although lung cancer is the leading cause of cancer-related death worldwide,^[1]^ the recent discovery of driver oncogene alterations such as epidermal growth factor receptor mutations or anaplastic lymphoma kinase rearrangements and the identification of their targeted inhibitors have dramatically improved outcomes in a subset of highly selected lung cancer patients.^[2–4]^ Consequently, the emergence of personalized therapies for non-small cell lung cancer underscores the need for cytologic or tissue verification of lung cancer. However, pre-operative biopsy can only obtain a limited tumor volume, and these small samples may lead to underestimation or misdiagnosis of complex intratumor heterogeneity.^[5–7]^

Imaging data could provide useful complementary information regarding the whole tumor.^[8,9]^ Computed tomography (CT) is fundamentally an anatomic imaging modality with rudimentary functional information available by comparing pre-contrast and post-contrast imaging.^[10]^ In addition, advances in other imaging methods, specifically positron-emission tomography-CT (PET-CT) for assessing tissue metabolism,^[10–12]^ and diffusion-weighted magnetic resonance imaging (DWI) reflecting cellular density and microstructural organization,^[13–15]^ can improve the accuracy of baseline staging compared with analysis by CT alone and can be used as surrogate biomarkers for cellularity or pathologic grade.^[16,17]^

The purpose of this article is to evaluate the relationship of functional imaging modality (PET-CT and DWI) with pathologic intratumor heterogeneity and cellularity in lung adenocarcinoma.

## 2 Material and methods

### 2.1 Patients

This study was performed as part of an ongoing prospective clinical trial seeking to determine the value of DWI and PET-CT findings compared with tumor pathologic grade and aggressiveness (NCT01585545).^[18]^ The study was approved by the institutional review board (SMC 2011-09-083), and written informed consent was obtained.

From November 2011 to December 2012, a total of 92 patients with operable lung adenocarcinoma were eligible for our study. The inclusion criteria of our study were as follows:

1. clinically and radiologically suspected lung adenocarcinoma,
2. clinically diagnosed stage I or II disease,
3. Eastern Cooperative Oncology Group performance status of 0 or 1 and eligible for surgery,
4. age 20 years or older, and
5. able to provide study-specific informed consent.

The exclusion criteria were:

1. non-adenocarcinoma lung disease (including benign disease, metastasis, non-adenocarcinoma lung cancer, and mucinous adenocarcinoma) and
2. not a candidate magnetic resonance (MR) imaging scan (poor renal function, metallic artifact, etc.).

Multiple studies indicate that mucinous adenocarcinoma is a variant of adenocarcinoma that has major clinical, radiologic, pathologic, and genetic differences from tumors formerly classified as nonmucinous bronchioloalveolar carcinoma (BAC).^[19]^ Based on these studies, we concluded that elimination of mucinous adenocarcinoma from the inclusion criteria is better for consistent analysis.

### 2.2 Imaging and analysis

CT images were obtained with the following parameters: detector collimation, 1.25 or 0.625 mm; 120 kVp; 150 to 200 mA; and reconstruction interval of 1 to 2.5 mm. All images were displayed at standard lung window settings (window width, 1500 HU; window level, −700 HU). All CT scans were obtained with 80cc of contrast material at 2cc/sec followed by normal saline 20cc at 2cc/sec. Two radiologists independently evaluated CT images on the viewing monitor of a picture archiving and communication system (GE Centricity v 2.1; GE). We regarded the consolidation component of the tumor as a solid portion and the ground-glass opacity (GGO) as a non-solid portion. The consolidation component was defined as an area of homogeneous increase in lung parenchymal attenuation that obscures the margins of vessels and airway walls. Lung lesions, composed of microscopic changes under CT resolution capability, manifest as GGOs (hazy increased opacity with preservation of bronchial and vascular margins on high-resolution computed tomography).

Before the curative operation, patients underwent CT and ^18^F-fluorodeoxyglucose (FDG) PET-CT examination. Before PET-CT examination, all patients fasted for at least six hours; after a normal blood glucose level in peripheral blood was ensured, patients received an intravenous injection of 379 MBq (10 mCi) of fluorine-18-FDG and rested for approximately 45 minutes before being scanned.^[20]^ Scans were acquired with a PET-CT device (Discovery LS; GE Medical Systems, Milwaukee, WI).

In addition, MRI was performed on a 1.5-T helium-cooled superconducting MRI scanner (Magnetom Avanto 1.5T, Siemens, Germany) with surface array coils and 32 receiving channels.^[21]^ Respiratory gated spectral *attenuated inversion recovery* fat suppressed single-shot echo planar DWI was obtained with b values of 0, 50, 100, 150, 250, 500, and 900 s/mm^2^. The parameters for DWI were TR, 11700 ms; TE, 73 ms; slice thickness, 5 mm; slice gap, 0.9 mm; matrix, 192 × 142; and field-of-view, 192 × 142 mm. Apparent diffusion coefficient (ADC) maps were automatically generated from DWIs obtained at b values of 0, 50, 100, 150, 250, 500, and 900 s/mm^2^ using standard post-processing software.

Semiautomatic drawing was used to delineate the corresponding lesion on T2-weighted axial imaging of the best spatial resolution as the reference point, which was copied to the corresponding ADC map. To ensure that the region of interest was placed within a tumor, a T2-weighted axial image and a CT axial image from PET-CT were aligned with each other. Then, semi-automatic drawing was performed for quantitative analysis of FDG uptake and ADC value. For semi-quantitative analysis of FDG uptake, a nuclear medicine physician, unaware of clinical information, placed regions of interest over the most intense area of FDG accumulation, which was interpreted as the maximum standardized uptake value (SUVmax). PET-CT imaging, MRI, and surgical resection were performed within 7 days of one another (mean interval, 3 days).

### 2.3 Pathologic evaluation and analysis

For tumor sampling, tumor tissues were taken from the resected tumor specimen at intervals of approximately 10 mm and placed on slides. All slides were scanned to produce high-resolution digital images (0.25 μm/pixel at 40 × magnification) using the Aperio Slide Scanning System (ScanScope T3; Aperio Technologies Inc., Vista, CA). Two experienced lung pathologists with 13 and 18 years of experience in lung pathology retrospectively interpreted the virtual slides using ImageScope viewing software (Aperio Technologies, Inc.) with a high-resolution monitor and measured the extent of invasive components and tumor cellularity (%).^[18,22]^

Histologic subtypes and grades of lung adenocarcinomas were classified according to the International Association for the Study of Lung Cancer/American Thoracic Society/European Respiratory Society (IASLC/ATS/ERS) multidisciplinary classification of lung adenocarcinomas.^[19]^ For each case, histologic subtyping was performed for the primary tumor in a semi-quantitative manner, with each histologic component (adenocarcinoma in situ [AIS], minimally invasive adenocarcinoma [MIA], lepidic predominant invasive adenocarcinoma, acinar-predominant invasive adenocarcinoma, papillary predominant invasive adenocarcinoma, micropapillary predominant invasive adenocarcinoma, solid predominant invasive adenocarcinoma, or variant type) accounted for in 5% increments, for a total of 100% for each tumor. Next, the most predominant pattern in mixed-type adenocarcinoma was defined as the histopathologic subtype that constituted the greatest percentage of the tumor. Predominant histologic subtypes were stratified into 3 pattern groups: AIS/MIA/lepidic, acinar/papillary, and micropapillary/solid groups. These pattern groups represent low, intermediate, and high grades of clinical behavior, respectively, based on an architectural grading system of histologic subtypes.^[23,24]^

### 2.4 Statistical analysis

Pearson correlation coefficient analysis and analysis of variance (ANOVA) were used to assess the relationships among pathologic parameters and imaging parameters. Imaging parameters were compared among the histologic grades (low, intermediate, and high) using one-way ANOVA with Bonferroni post hoc test.

Statistical significance was evaluated with software (SPSS, version 19.0, 2010; SPSS, Chicago, IL). A *P* value less than .05 was considered statistically significant.

## 3 Results

### 3.1 Clinical characteristics of patients and tumors

**Figure 1:**
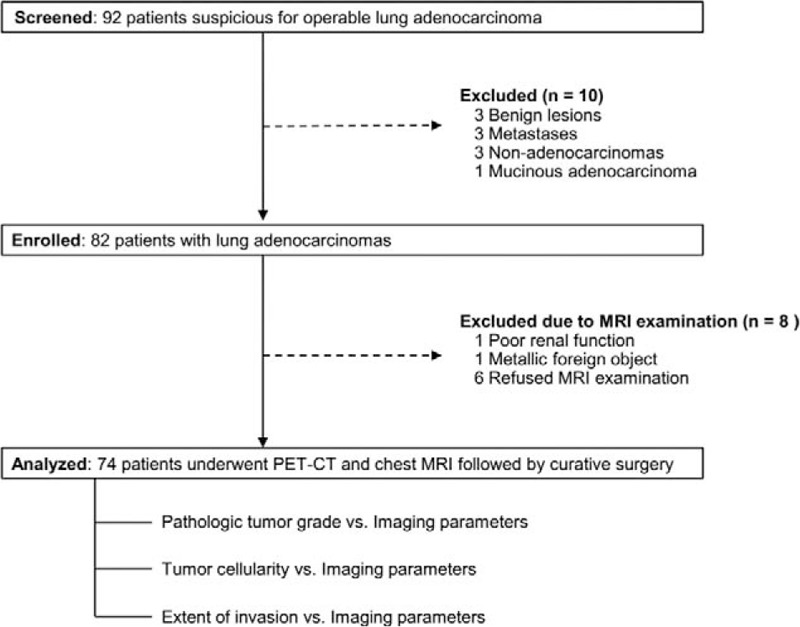
Flow diagram of the patient cohort.

In total, 74 early-stage lung adenocarcinomas with preoperative chest MRI and PET-CT were included in our study (Fig. 1). The detailed clinicopathologic characteristics of the 74 lung adenocarcinomas are summarized in Tables 1 and 2.

**Table 1:**
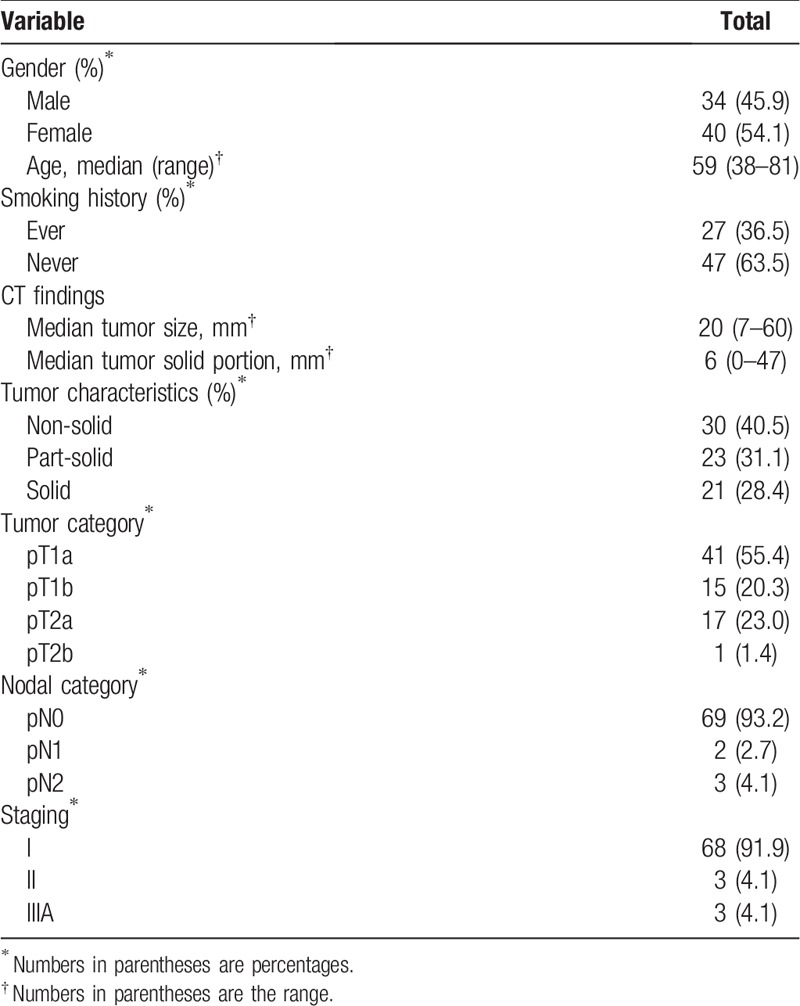
Demographic characteristics of lung adenocarcinoma cases.

**Table 2:**
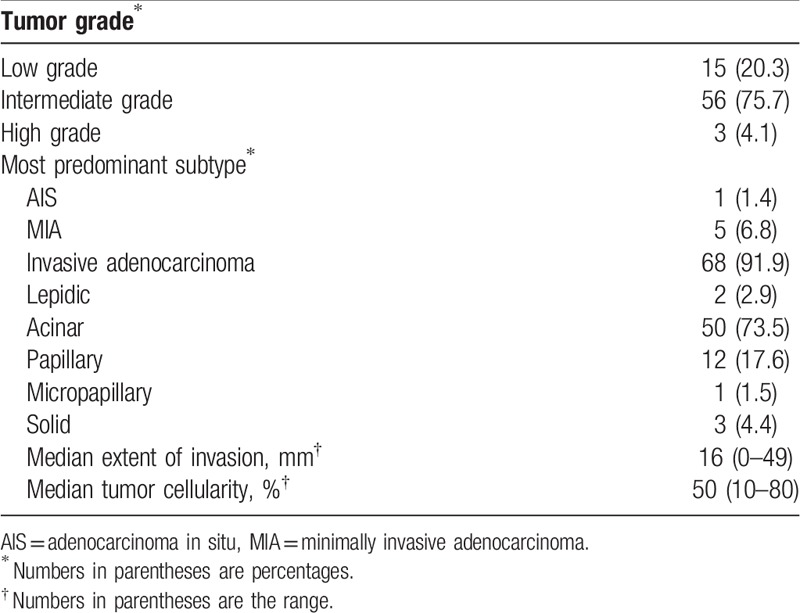
Pathologic characteristics of lung adenocarcinoma tumors.

Final pathologic staging revealed 68 stage I tumors (91.9%), 3 stage II tumors (4.1%), and 3 stage IIIA tumors (4.1%) (Table 1). The most frequent tumor histologic grade was intermediate grade (76%), followed by low grade (20%) and high grade (4%). The most predominant histologic subtype was the acinar subtype, followed by the papillary subtype (Table 2).

### 3.2 Correlations between pathologic grade and imaging biomarkers

The relationships among pathologic grade, tumor size, and imaging biomarkers are described in Table 3. Solid tumor size and SUVmax had a positive relationship according to pathologic grade, the differences among which were significant (*P* = .01 and *P* < .01, respectively). The ADC from DWI showed an inverse relationship with pathologic grade (low: 1.30 ± 0.17, intermediate: 1.13 ± 0.22, and high: 1.05 ± 0.14).

**Table 3:**
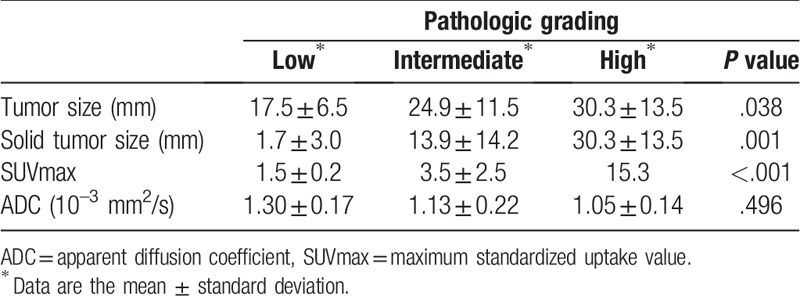
Comparison of pathologic grade and imaging factors.

### 3.3 Comparison between pathologic tumor invasion and imaging biomarkers

**Figure 2:**
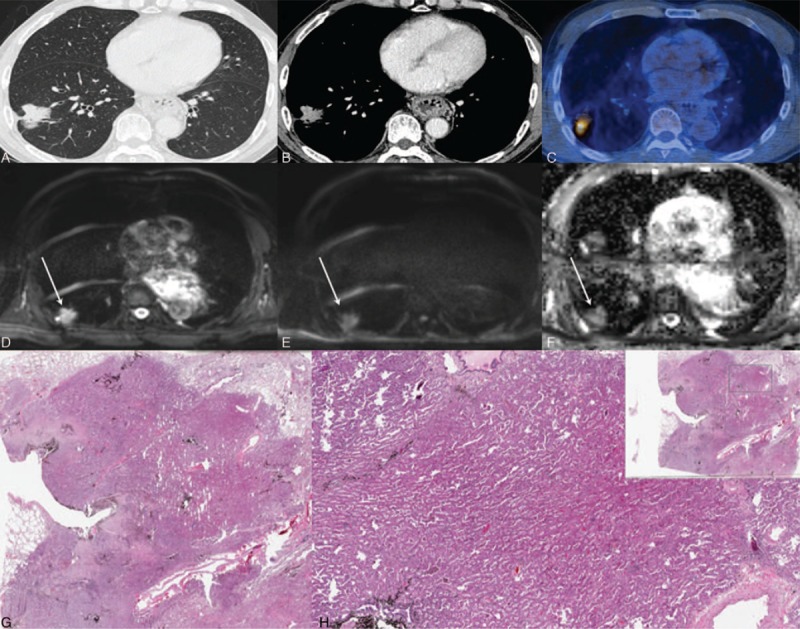
A 61-year-old man with lung adenocarcinoma (tumor cellularity: 80%). Chest CTs on the (A) lung setting and (B) mediastinal setting show a well-defined enhancing nodule in the right lower lobe. (C) PET-CT shows increased FDG uptake (SUVmax = 15.4). (D) Low DWI (b = 0), (E) high DWI (b = 900), and (F) ADC demonstrate restricted diffusion within this lesion (ADC value = 752 × 10^–6^ mm^2^/s). (G) H&E staining (magnification = 2 × ) and (H) H&E staining (magnification = 20×) show the area at the corresponding site. Arrows (D–F) indicate lung cancer. H&E = hematoxylin and eosin.

**Figure 3:**
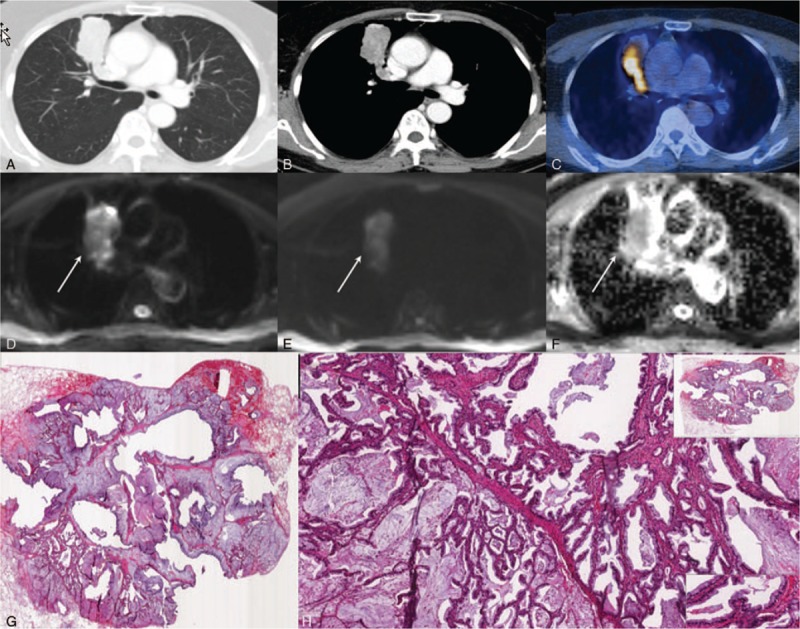
A 65-year-old woman with lung adenocarcinoma (tumor cellularity: 20%). Chest CTs on the (A) lung setting and (B) mediastinal setting show a well-defined heterogeneously enhancing mass in the right upper lobe. (C) PET-CT shows increased FDG uptake (SUVmax = 4.4). (D) Low DWI (b = 0), (E) high DWI (b = 900), and (F) ADC demonstrate restricted diffusion within this lesion (ADC value = 1560 × 10^–6^ mm^2^/s). (G) H&E staining (magnification = 2×) and (H) H&E staining (magnification = 20×) show the area at the corresponding site. Arrows (D–F) indicate lung cancer. H&E = hematoxylin and eosin.

**Figure 4:**
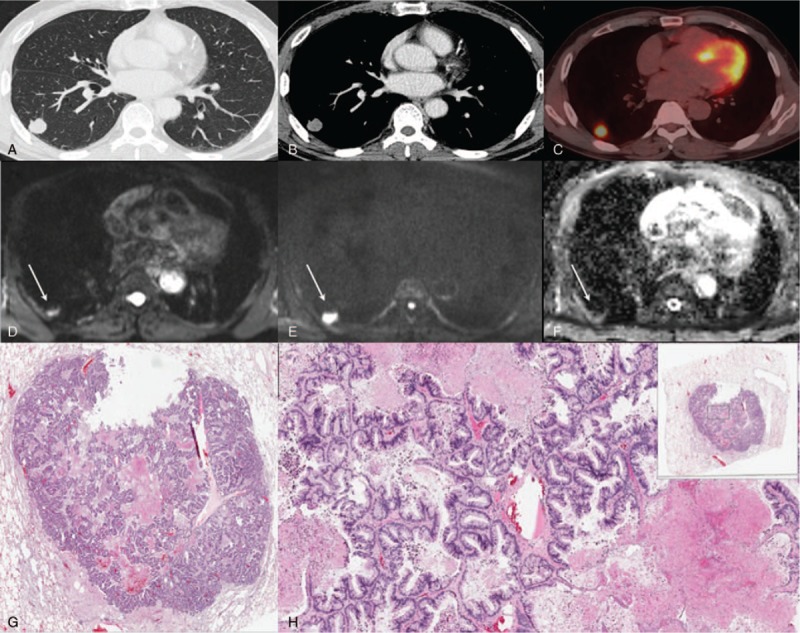
A 62-year-old man with lung adenocarcinoma (tumor cellularity: 80%). Chest CTs on the (A) lung setting and (B) mediastinal setting show a well-defined enhancing nodule in the right lower lobe. (C) PET-CT shows increased FDG uptake (SUVmax = 9.0). (D) Low DWI (b = 0), (E) high DWI (b = 900), and (F) ADC demonstrate restricted diffusion within this lesion (ADC value = 1092 × 10^–6^ mm^2^/s). (G) H&E staining (magnification = 2×) and (H) H&E staining (magnification = 20×) show the area at the corresponding site. Arrows (D–F) indicate lung cancer. H&E = hematoxylin and eosin.

**Figure 5:**
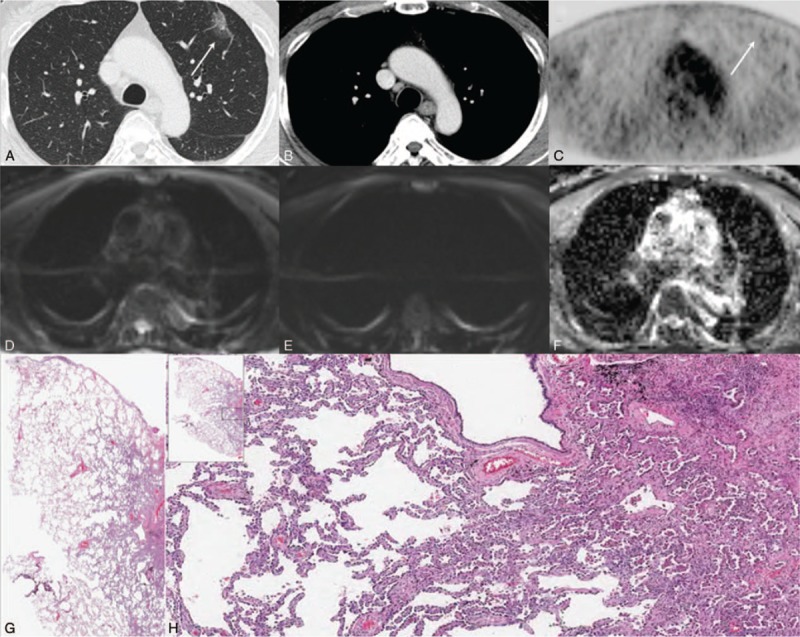
A 64-year-old man with lung adenocarcinoma (tumor cellularity: 70%). Chest CTs on the (A) lung setting and (B) mediastinal setting show a well-defined GGO lesion in the left upper lobe. (C) PET-CT shows faint FDG uptake (SUVmax = 9.4). (D) Low DWI (b = 0), (E) high DWI (b = 900), and (F) ADC demonstrate slightly restricted diffusion within this lesion (ADC value = 559 × 10^–6^ mm^2^/s). (G) H&E staining (magnification = 2×) and (H) H&E staining (magnification = 20×) show the area at the corresponding site. Arrows (A and C) indicate lung cancer. GGO = ground glass opacity; H&E = hematoxylin and eosin.

The relationships among extent of pathologic invasion, tumor cellularity, and imaging biomarkers are described in Table 4 (Figs. 2–5). The mean diameter of pathologic invasion ranged from 0 to 49 mm (median: 16 mm). The mean percentage of tumor cellularity in all adenocarcinomas ranged from 10 to 80% (median: 50%). SUVmax showed a strong correlation with tumor cellularity (*R* = 0.713, *P* < .001), but was not correlated with extent of invasion (*R* = 0.387, *P* = .148). *In addition, the 3 other parameters (total* tumor size, solid tumor size, and ADC) *were* nonsignificant *with P values of .715, .789, and .065, respectively.*

**Table 4:**
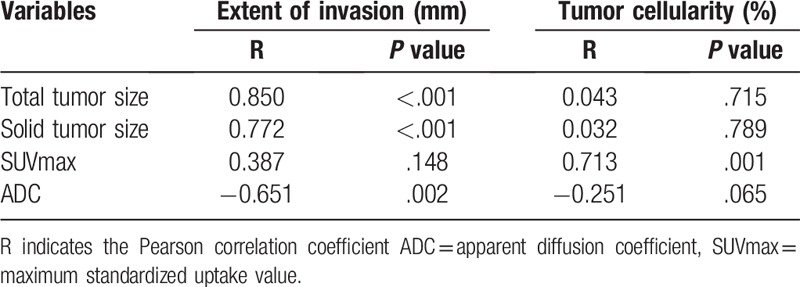
Comparison of extent of invasion and tumor cellularity with imaging factors.

## 4 Discussion

In our analysis, trends were observed toward higher SUVmax and lower ADC values in higher tumor cellularity or pathologic grade tumors compared with lower tumor cellularity or pathologic grade tumors. The relationship between SUVmax and tumor cellularity was statistically significant, while the ADC value did not exhibit a significant relationship with tumor cellularity.

Several studies have shown that SUVmax on FDG-PET correlates with the IASLC/ATS/ERS classification of lung cancer. In a previous work by Nakamura et al,^[25]^ SUVmax was a useful indicator of the malignant grade of each lung adenocarcinoma subtype. In this study, lung adenocarcinoma subtypes were classified into 3 subgroups (group A, AIS + MIA + lepidic predominant invasive adenocarcinoma [low risk]; group B, acinar predominant invasive adenocarcinoma + papillary predominant invasive adenocarcinoma + invasive mucinous adenocarcinoma [intermediate risk]; and group C, solid predominant invasive adenocarcinoma + micropapillary predominant invasive adenocarcinoma [high risk]), which are similar to our 3 pathologic grades. SUVmax was lower in group A, intermediate in group B, and higher in group C. Significant differences in SUVmax among the subgroups were detected (*P* < .0001). Kadota et al^[26]^ also found a positive association between SUVmax and histologic grade. Tumors with high-grade histology had a higher SUVmax (6.2 ± 2.8) than those with intermediate-grade (3.7 ± 2.5) or low-grade (2.5 ± 1.6) histology (*P* < .001).

According to a meta-analysis by Chen et al,^[27]^ ADC had a moderate inverse correlation with cellularity among various tumor types. In this study, ADC correlated strongly with cell count in lung cancer (*P* = −.63, 95% CI = [−0.78, −0.48]), although only a small number of patients were included (69 patients, 4.51%). Based on the reported data, it has been postulated that DWI, namely ADC, can be an imaging tool for estimating tumor cellularity.^[28]^ However, there were also reports in which no significant correlations between ADC and cell count were found.^[29,30]^

These statistically discrepant correlations in SUVmax and ADC with tumor cellularity might be explained in part by the concept of tumor microenvironment, where the tumor volume consists of a real tumor component as well as a non-tumorous stromal component. The stromal area consists of extracellular matrix components and several cell types, including cancer-associated fibroblasts, immune cells, vascular cells, and bone marrow-derived cells,^[31–34]^ which generally have a greater extracellular volume compared with tumors with dense cellularity.^[34,35]^ A very strong interdependence between areas of nuclei and stroma was seen, which indicates that cellularity and stromal area were indistinguishable and interchangeable. Tumor cellularity in our study was based on visual assessment by two pathologists, who evaluated only tumor cells and excluded stromal non-tumorous cells. Although DWI is well-known to reflect cellularity, measurement from DWI consists of tumor cells and even stromal non-tumorous cells. Consequently, ADC values may not match tumor cellularity assessments by pathologists. Meanwhile, SUVmax reflects tumor cells more than whole tumor microenvironments including stromal cells. Therefore, we comprehensively evaluated the tumor microenvironment of tumor cells as well as non-tumorous stromal cells by considering both DWI and PET information.

Traditionally, histologic subtyping and subsequent tumor scoring or grading for lung adenocarcinomas are estimated using a resected surgical specimen (whole tumor), not by using a core biopsy or cytologic material. However, in cases of unresectable lung cancer, histopathologic information is solely based on small specimens obtained via tumor biopsy. Thus, this method is both subjective and inevitably prone to errors related to intratumor heterogeneity. In addition, approximately 80% of patients with lung cancer have an unresectable tumor at the time of initial presentation.^[36]^ Therefore, it is desirable to predict patient prognosis using surrogate imaging biomarkers that could reflect histopathologic stratification.^[37]^ Quantifying the disparity between PET assessing tissue metabolism, and DWI reflecting whole cellular density, could be a better representation of purely tumor cellular activity, but not including stromal areas.^[38]^

Our study had several limitations. First, there were a small number of subjects included in our study, and they were enrolled from a single institution. In particular, results from small numbers of certain subtypes of adenocarcinoma may not be generalizable to other subtypes. Second, there was the potential for disagreement between the pathologists making pathologic evaluations. However, an effort was made to minimize disagreement, since pathologic assessment was the standard of reference in our study. Two pathologists reviewed each case independently, followed by reaching a consensus in a 2-step order. In addition, a digital microscope system was used to achieve objective visual assessment ^[18]^. Third, there is a possibility that an inflammatory or infectious process could occur within the tumor, which is a limitation to accurately measuring tumor cellularity from imaging. In cases where better distinctions between malignant and non-malignant extent are needed, other PET-CT parameters like total lesion glycolysis (TLG) or PET tracers such as 18F-fluorothymidine (18F-FLT), which reflects cellular proliferation rather than less specific increased glucose metabolism, may be useful. However, these methods are not commonly used in daily medical practice and their availability for rapid clinical application is likely to be limited. Also, our correlation results for tumor cellularity and SUVmax were found to be meaningful and easily- applicable through retrospective pathology comparisons.

## 5 Conclusion

Despite significant progress in cancer diagnostics and the development of novel therapeutic regimens, the successful treatment of advanced forms of cancer is still a challenge that may require personalized therapeutic approaches. Metabolically active tumor burden provides a more complete estimation of biological aggressiveness and can serve as a complementary quantitative prognostic measure. Also, incorporating metabolic tumor burden in trials and staging could help subselect patient groups that would most benefit from adjuvant or neoadjuvant chemotherapeutic therapies. More accurate risk stratification may help clinicians and patients select optimal treatment and improve outcome prediction.

## Acknowledgments

The authors would like to offer special thanks to Insuk Sohn, PhD (Statistics and Data Center, Research Institute for Future Medicine, Samsung Medical Center, Seoul, Korea) for his assistance with the statistics used in this report.

## Author contributions

**Conceptualization:** Ho Yun Lee, Joon Young Choi.

**Data curation:** Joon Young Choi, O Jung Kwon, Hong Kwan Kim, Young Mog Shim.

**Funding acquisition:** Ho Yun Lee.

**Project administration:** Ho Yun Lee.

**Supervision:** Ho Yun Lee.

**Validation:** Ho Yun Lee.

**Writing – original draft:** Ki Hwan Kim, Seong-Yoon Ryu.

**Writing – review & editing:** Ki Hwan Kim, Seong-Yoon Ryu, Ho Yun Lee, Joon Young Choi, O Jung Kwon, Hong Kwan Kim, Young Mog Shim.
